# Supplementary material for: Genome-resolved metagenomics reveals nonmethanogenic soil lineages in an Andean páramo wetland
Source: ISME Commun. 2026 Oct 11;6(1):ycag294. doi: 10.1093/ismeco/ycag294 (PMC13730134; doi:10.1093/ismeco/ycag294)
Supplement: Supplementary file 1 [file supplementary_material_ycag294.zip › SupplFig1_ecosites.pdf]

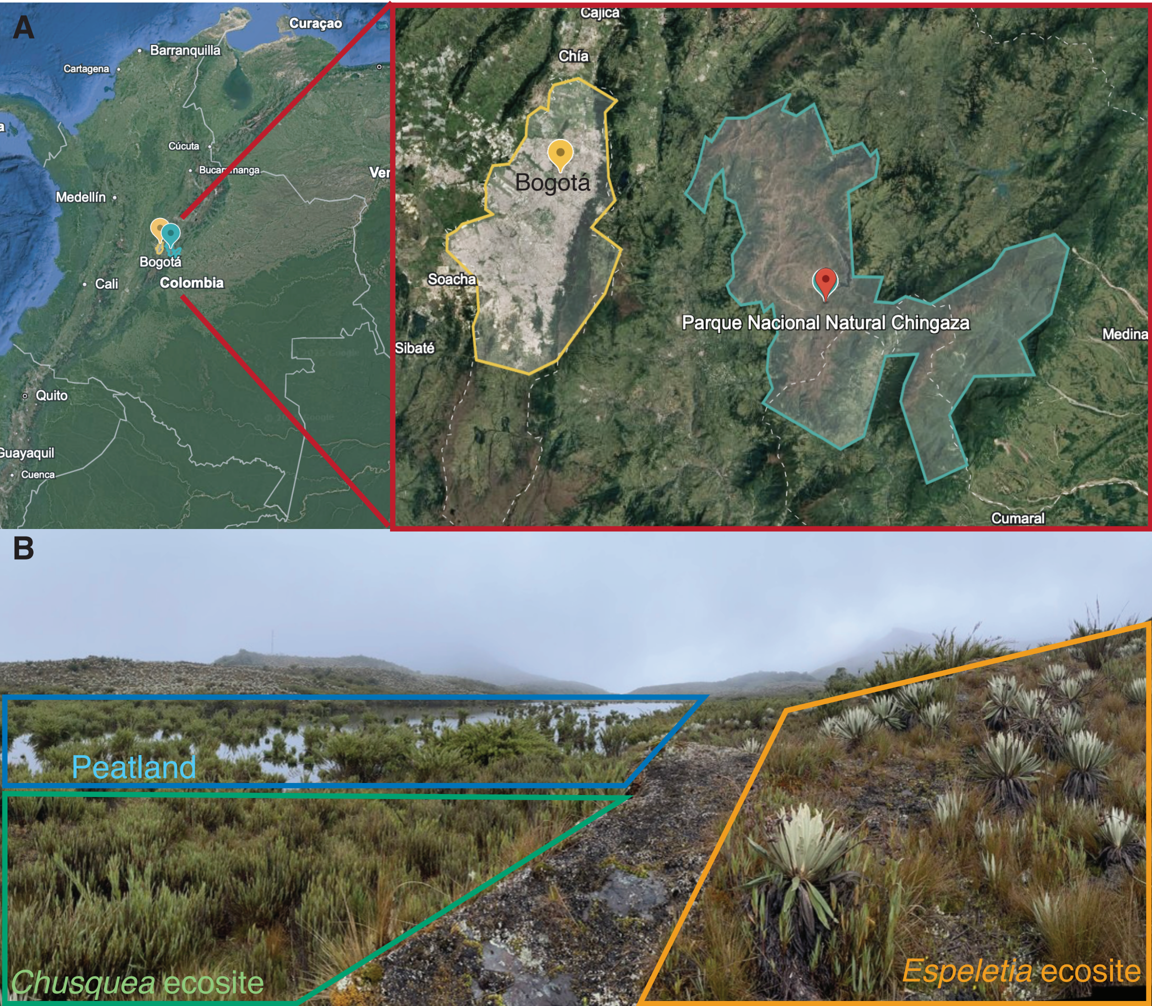

A
Curaçao
Barranquilla
Cartagena
Cúcuta
Medellín
Bogotá
Cali
Colombia
Quito
Guayaquil
Cuenca
Cajicá
Chía
Bogotá
Soacha
Sibaté
Parque Nacional Natural Chingaza
Medina
Cumaral
B
Peatland
Chusquea ecosite
Espeletia ecosite
