# Supplementary material for: Genome-resolved metagenomics reveals nonmethanogenic soil lineages in an Andean páramo wetland
Source: ISME Commun. 2026 Oct 11;6(1):ycag294. doi: 10.1093/ismeco/ycag294 (PMC13730134; doi:10.1093/ismeco/ycag294)
Supplement: Supplementary file 1 [file supplementary_material_ycag294.zip › SupplFig2_Combined_ShannonBox_Phylum_Aligned.pdf]

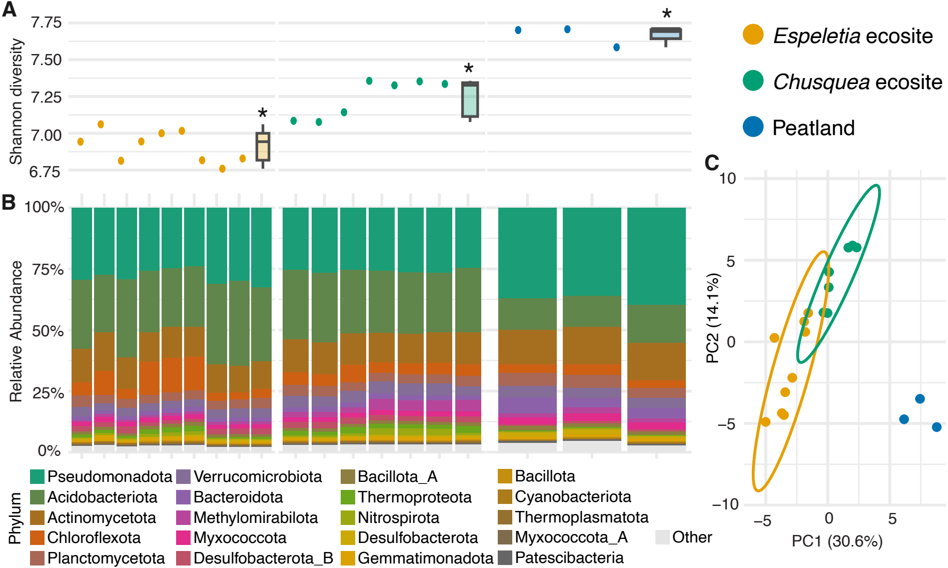

A
Shannon diversity
7.75
7.50
7.25
7.00
6.75
*
*
*
Espeletia ecosite
Chusquea ecosite
Peatland
B
100%
75%
50%
25%
0%
Relative Abundance
Phylum
Pseudomonadota
Acidobacteriota
Actinomycetota
Chloroflexota
Planctomycetota
Verrucomicrobiota
Bacteroidota
Methylomirabilota
Myxococcota
Desulfobacterota_B
Bacillota_A
Thermoproteota
Nitrospirota
Desulfobacterota
Gemmatimonadota
Bacillota
Cyanobacteriota
Thermoplasmatota
Myxococcota_A
Patescibacteria
Other
C
10
5
0
−5
−10
PC2 (14.1%)
−5
0
5
PC1 (30.6%)
