# Supplementary material for: Genome-resolved metagenomics reveals nonmethanogenic soil lineages in an Andean páramo wetland
Source: ISME Commun. 2026 Oct 11;6(1):ycag294. doi: 10.1093/ismeco/ycag294 (PMC13730134; doi:10.1093/ismeco/ycag294)
Supplement: Supplementary file 1 [file supplementary_material_ycag294.zip › SupplFig3_SharedRichness_Venn.pdf]

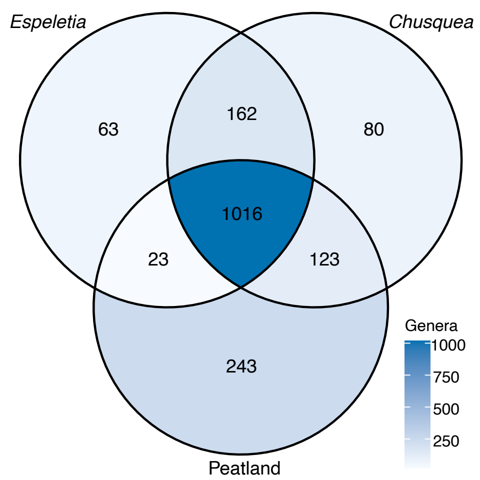

Espeletia
Chusquea
63
162
80
1016
23
123
243
Peatland
Genera
1000
750
500
250
