# Supplementary material for: Genome-resolved metagenomics reveals nonmethanogenic soil lineages in an Andean páramo wetland
Source: ISME Commun. 2026 Oct 11;6(1):ycag294. doi: 10.1093/ismeco/ycag294 (PMC13730134; doi:10.1093/ismeco/ycag294)
Supplement: Supplementary file 1 [file supplementary_material_ycag294.zip › SupplFig4_quality_assessment.pdf]

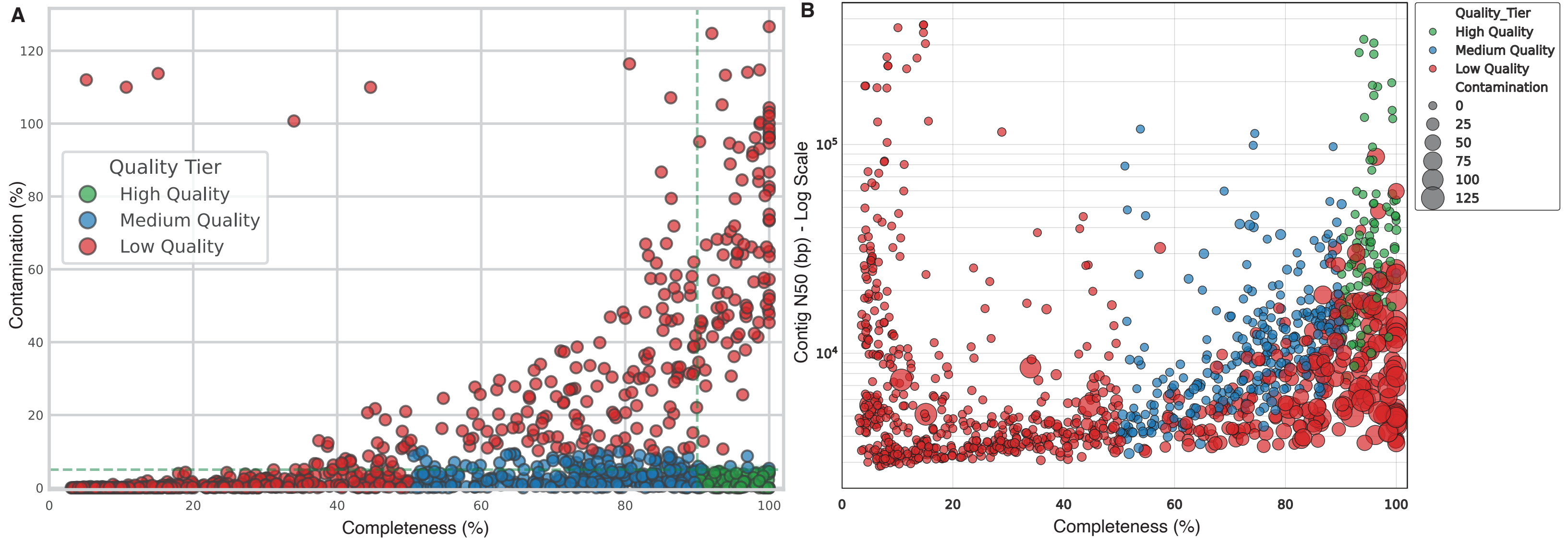

A
Contamination (%)
Completeness (%)
Quality Tier
High Quality
Medium Quality
Low Quality
0
20
40
60
80
100
120
B
Contig N50 (bp) - Log Scale
10^5
10^4
Quality_Tier
Contamination
25
50
75
125
