# Supplementary material for: Genome-resolved metagenomics reveals nonmethanogenic soil lineages in an Andean páramo wetland
Source: ISME Commun. 2026 Oct 11;6(1):ycag294. doi: 10.1093/ismeco/ycag294 (PMC13730134; doi:10.1093/ismeco/ycag294)
Supplement: Supplementary file 1 [file supplementary_material_ycag294.zip › SupplFig5_redundancy.pdf]

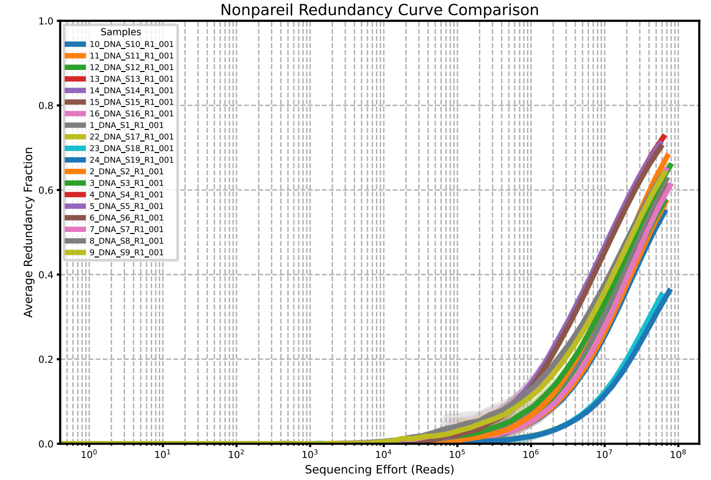

Nonpareil Redundancy Curve Comparison
Samples
10_DNA_S10_R1_001
11_DNA_S11_R1_001
12_DNA_S12_R1_001
13_DNA_S13_R1_001
14_DNA_S14_R1_001
15_DNA_S15_R1_001
16_DNA_S16_R1_001
1_DNA_S1_R1_001
22_DNA_S17_R1_001
23_DNA_S18_R1_001
24_DNA_S19_R1_001
2_DNA_S2_R1_001
3_DNA_S3_R1_001
4_DNA_S4_R1_001
5_DNA_S5_R1_001
6_DNA_S6_R1_001
7_DNA_S7_R1_001
8_DNA_S8_R1_001
9_DNA_S9_R1_001
Average Redundancy Fraction
0.0
0.2
0.4
0.6
0.8
1.0
Sequencing Effort (Reads)
10^0
10^1
10^2
10^3
10^4
10^5
10^6
10^7
10^8
