# Supplementary material for: Genome-resolved metagenomics reveals nonmethanogenic soil lineages in an Andean páramo wetland
Source: ISME Commun. 2026 Oct 11;6(1):ycag294. doi: 10.1093/ismeco/ycag294 (PMC13730134; doi:10.1093/ismeco/ycag294)
Supplement: Supplementary file 1 [file supplementary_material_ycag294.zip › SupplFig6_MAG_recruitment.pdf]

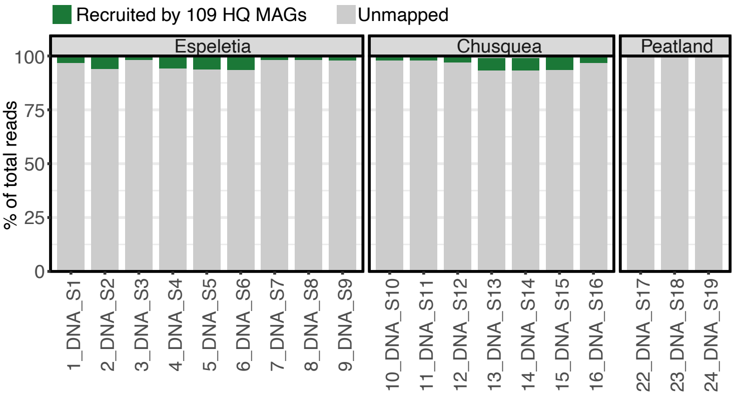

Recruited by 109 HQ MAGs
Unmapped
Espeletia
Chusquea
Peatland
% of total reads
0
25
50
75
100
1_DNA_S1
2_DNA_S2
3_DNA_S3
4_DNA_S4
5_DNA_S5
6_DNA_S6
7_DNA_S7
8_DNA_S8
9_DNA_S9
10_DNA_S10
11_DNA_S11
12_DNA_S12
13_DNA_S13
14_DNA_S14
15_DNA_S15
16_DNA_S16
22_DNA_S17
23_DNA_S18
24_DNA_S19
