# Supplementary material for: Genome-resolved metagenomics reveals nonmethanogenic soil lineages in an Andean páramo wetland
Source: ISME Commun. 2026 Oct 11;6(1):ycag294. doi: 10.1093/ismeco/ycag294 (PMC13730134; doi:10.1093/ismeco/ycag294)
Supplement: Supplementary file 1 [file supplementary_material_ycag294.zip › SupplFig7_tree_pruned_references.pdf]

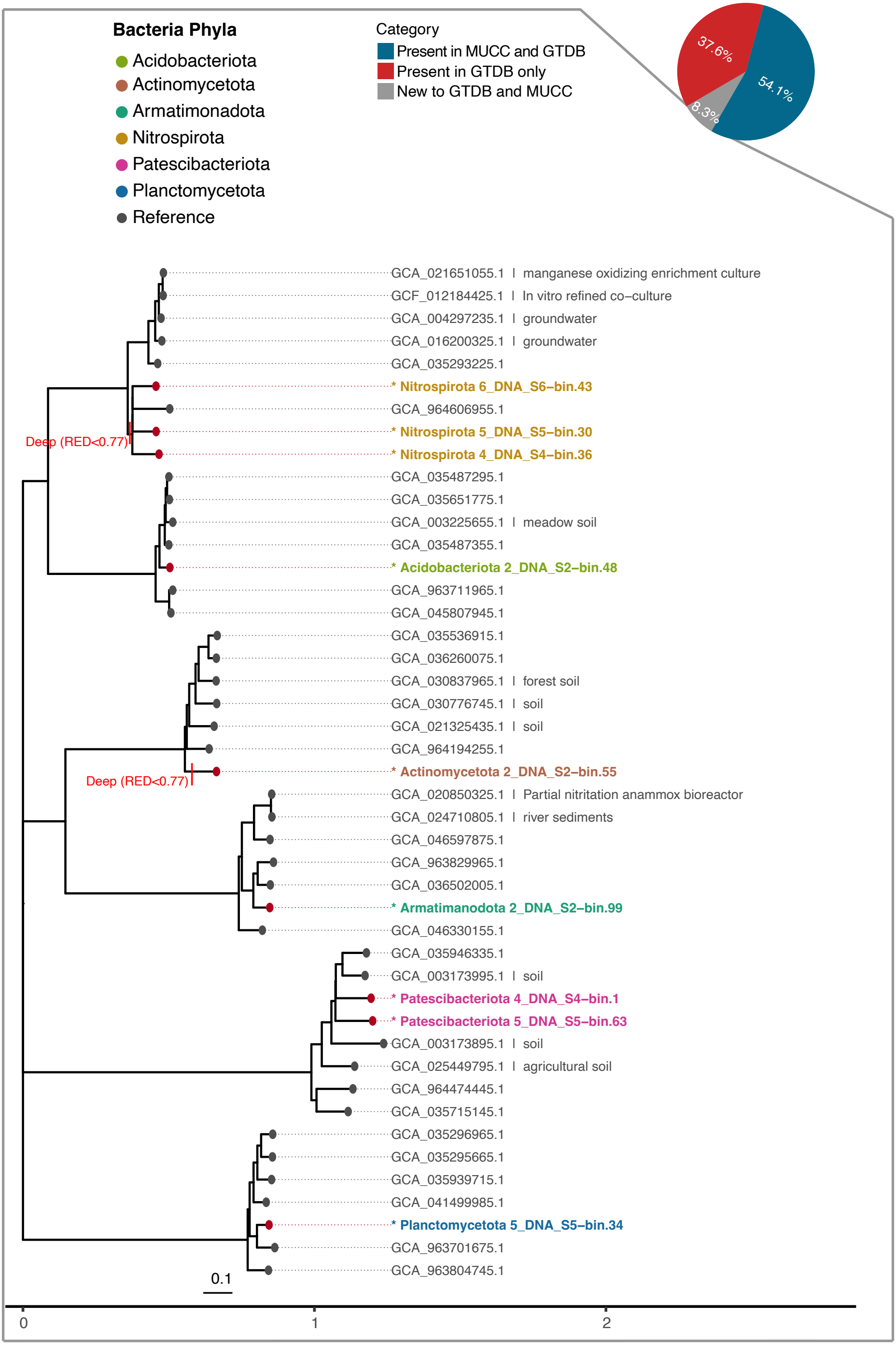
Bacteria Phyla
Acidobacteriota
Actinomycetota
Armatimonadota
Nitrospirota
Patescibacteriota
Planctomycetota
Reference
Category
Present in MUCC and GTDB
Present in GTDB only
New to GTDB and MUCC
37.6%
54.1%
8.3%
GCA_021651055.1 | manganese oxidizing enrichment culture
GCF_012184425.1 | In vitro refined co–culture
GCA_004297235.1 | groundwater
GCA_016200325.1 | groundwater
GCA_035293225.1
* Nitrospirota 6_DNA_S6–bin.43
GCA_964606955.1
* Nitrospirota 5_DNA_S5–bin.30
* Nitrospirota 4_DNA_S4–bin.36
Deep (RED<0.77)
GCA_035487295.1
GCA_035651775.1
GCA_003225655.1 | meadow soil
GCA_035487355.1
* Acidobacteriota 2_DNA_S2–bin.48
GCA_963711965.1
GCA_045807945.1
GCA_035536915.1
GCA_036260075.1
GCA_030837965.1 | forest soil
GCA_030776745.1 | soil
GCA_021325435.1 | soil
GCA_964194255.1
* Actinomycetota 2_DNA_S2–bin.55
Deep (RED<0.77)
GCA_020850325.1 | Partial nitritation anammox bioreactor
GCA_024710805.1 | river sediments
GCA_046597875.1
GCA_963829965.1
GCA_036502005.1
* Armatimanodota 2_DNA_S2–bin.99
GCA_046330155.1
GCA_035946335.1
GCA_003173995.1 | soil
* Patescibacteriota 4_DNA_S4–bin.1
* Patescibacteriota 5_DNA_S5–bin.63
GCA_003173895.1 | soil
GCA_025449795.1 | agricultural soil
GCA_964474445.1
GCA_035715145.1
GCA_035296965.1
GCA_035295665.1
GCA_035939715.1
GCA_041499985.1
* Planctomycetota 5_DNA_S5–bin.34
GCA_963701675.1
GCA_963804745.1
0.1
0
1
2
